# Bacterial contributions to cancer development: mechanisms, dysbiosis, and cross-cancer associations

**DOI:** 10.1186/s13027-025-00722-7

**Published:** 2026-01-08

**Authors:** Yasmin N. Ramadan, Marah N. Alatawi, Abdullah S. Albalawi, Helal F. Hetta

**Affiliations:** 1 Department of Microbiology and Immunology, Faculty of Pharmacy, Assiut University, Assiut, 71515 Egypt; 2 Department of Medical Laboratory Technology, Faculty of Applied Medical Sciences, University of Tabuk, Tabuk, 71491 Saudi Arabia; 3 Department of Pharmaceutical Chemistry, Faculty of Pharmacy, University of Tabuk, Tabuk, 71491 Saudi Arabia; 4 Division of Microbiology, Immunology and Biotechnology, Department of Natural Products and Alternative Medicine, Faculty of Pharmacy, University of Tabuk, Tabuk, 71491 Saudi Arabia

**Keywords:** Oncogenic bacteria, carcinogenesis, Molecular mechanisms, Microbiome dysbiosis, Cancer-associated microbiota, Therapeutic insights

## Abstract

Cancer is a multifactorial disease influenced by complex interactions between genetic, environmental, and microbial factors. Growing evidence demonstrates that several bacterial species contribute to carcinogenesis through chronic inflammation, production of genotoxic metabolites, disruption of cell-cycle regulation, and modulation of host immune responses. *Helicobacter pylori*–induced gastric adenocarcinoma remains the most well-established example of bacteria-driven oncogenesis; however, accumulating studies indicate that additional virulence mechanisms and bacteria-dependent pathways are also implicated in tumor initiation and progression across multiple organ systems. Given the increasing global burden of cancer and rising concerns regarding antimicrobial resistance, understanding the oncogenic roles of bacteria has become essential for guiding prevention and therapeutic strategies. This review provides a novel and integrated perspective by synthesizing mechanistic, microbiome-driven, and cross-cancer evidence on how bacterial pathogens contribute to oncogenesis, while also highlighting the shared microbial patterns observed in both gastrointestinal and extraintestinal cancers such as breast cancer. In this mini-review, we summarize the molecular mechanisms through which oncogenic bacteria facilitate cancer development, discuss microbiome dysbiosis associated with various malignancies, and examine their roles in gastric, colorectal, esophageal, lung, gallbladder, breast, and cervical cancers. Understanding these interconnected pathways may support future development of microbiome-targeted therapeutic interventions and facilitate early detection strategies.

## Introduction

Cancer remains the second leading cause of mortality worldwide and represents a complex, multifactorial disease characterized by the accumulation of genetic, metabolic, and immunological abnormalities. Classical hallmarks of cancer, including sustained proliferation, evasion of growth suppressors, resistance to apoptosis, angiogenesis, replicative immortality, and metastatic potential, are driven by genomic instability and chronic inflammation, which serve as underlying enabling factors. Advances in molecular oncology continue to refine our understanding of the diverse contributors to tumor initiation and progression [1–3].

Global projections emphasize the urgency of improved prevention and management: the World Health Organization estimates ~20 million new cancer cases in 2022 with a projected rise to ~ 35 million by 2050, driven in part by tobacco, alcohol, obesity and environmental factors [4]. According to IARC's Global Cancer Observatory, ten cancer types accounted for over two-thirds of new cases in 2022; lung (≈2.5 million), female breast (≈2.3 million), colorectal (≈1.9 million), prostate (≈1.5 million) and stomach (≈970,000) were among the most frequent [5].

**Fig. 1:**
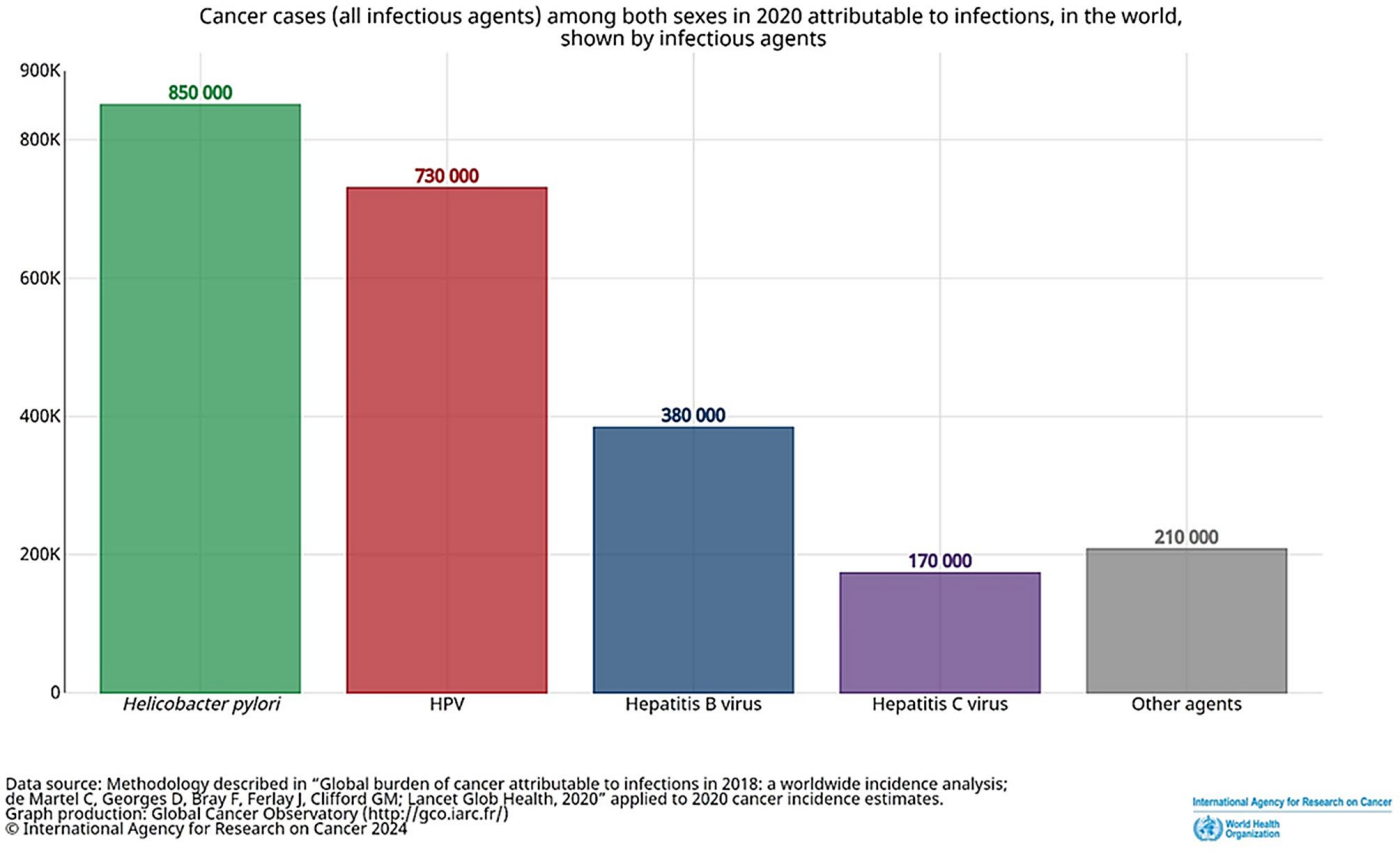
Infection-related global cancer burden and major microbial contributors. Summary of the estimated global cancer cases attributable to infectious agents in 2020, based on recent IARC data. *H. pylori* represents the leading cause of infection-associated cancers (~850,000 cases), followed by human papillomavirus (~730,000 cases), hepatitis B (~380,000 cases), and hepatitis C (~170,000 cases). The figure illustrates the disproportionate burden caused by bacterial and viral pathogens and provides epidemiological context for understanding how microbial infections—including established bacteria-linked malignancies—contribute to cancer development worldwide [7, 8]

Infectious agents are an established contributor to the global cancer burden. Current estimates attribute roughly 15–20% of cancers to infections, with several agents classified as carcinogenic by IARC [6; 7]. Notably, *Helicobacter pylori* accounted for an estimated 850,000 infection-attributable cancer cases in 2020, followed by human papillomavirus (≈730,000), and hepatitis B and C viruses (≈380,000 and ≈170,000, respectively) [8] (Fig. 1). While viral oncogenesis is well characterized—largely through viral integration and direct genetic interference (de Oliveira et al. 2016)—accumulating epidemiological and mechanistic evidence highlights a substantive role for bacteria in carcinogenesis [9; 10].

Bacteria may contribute to cancer by multiple, often interconnected mechanisms. Persistent infection and chronic inflammation create a tumor-promoting microenvironment through cytokine release, reactive oxygen/nitrogen species (ROS/RNS) generation, and immune dysregulation. Bacterial toxins and genotoxins (e.g., colibactin, cytolethal distending toxin) directly damage host DNA or interfere with cell-cycle checkpoints, while bacterial effectors can hijack signaling pathways such as NF-κB, STAT3, Wnt/β-catenin, and PI3K/Akt to promote proliferation and survival [11–14]. In addition, microbiome dysbiosis alters metabolic outputs—including short-chain fatty acids, secondary bile acids, and estrogen-metabolizing enzymes—that can act systemically to modulate tumor risk in extraintestinal sites such as the breast [15]. Representative clinical associations include *H. pylori* with gastric adenocarcinoma and MALT lymphoma, *Salmonella Typhi* with gallbladder carcinoma, and specific strains of *Escherichia coli*, *Bacteroides fragilis*, and *Fusobacterium* spp. with colorectal cancer [6, 7, 16].

Despite growing data, the bacterial contribution to cancer remains incompletely defined: causality is established for relatively few microbes, and mechanistic links vary by pathogen, host context, and microbiome composition. Advances in high-throughput sequencing and functional microbiology have begun to reveal tumor-associated microbial communities and their interactions with the immune system and epithelial signaling; however, a comprehensive synthesis that integrates mechanistic pathways with cross-cancer microbiome signatures is still needed.

The present review provides an updated, integrative synthesis of the molecular, immunological, and metabolic mechanisms by which bacteria contribute to cancer development across multiple organ systems. By linking mechanistic evidence with microbiome-associated dysbiosis patterns and comparing shared bacterial signatures across gastrointestinal and extra-intestinal malignancies (including the emerging gut–breast axis), this review offers a novel multidimensional perspective that advances beyond prior, more narrowly focused reports.

In this mini-review, we summarize key bacterial oncogenic mechanisms, discuss dysbiosis and microbial signatures associated with gastric, colorectal, esophageal, lung, gallbladder, breast, and cervical cancers, and outline implications for diagnostics and microbiome-targeted therapeutics.

## Oncogenic bacteria

A considerable approximately 25% of all cancer cases have been definitively associated with certain viral or microbial infections; however, these malignancies are primarily caused by a small number of viruses, such as hepatitis B and C viruses (for hepatocellular carcinoma), Epstein–Barr virus (for lymphoma), human papillomavirus (for cervical cancer, head and neck cancer squamous cell carcinomas), and human T cell lymphotropic viruses (for lymphoma and leukemia), and principally by one bacterium *H. pylori* (for gastric cancer) [7] (Fig. 1).

The gastrointestinal tract is the most microbially densely populated area of the human body, with the highest microbial densities seen in the colon [17]. Dysbiosis is associated with both systemic and gut-localized illnesses and has the ability to modify intestinal homeostasis. Furthermore, the complex networks between the microbiome and the host are represented by bidirectional crosstalk and several studies indicate that the development of various cancer types is influenced by microbiome dysbiosis [6, 18, 19], as summarized in Table 1. Preclinical studies during the past 20 years have contributed to the understanding of the mechanisms through which different types of bacteria, that are resident in human tumor tissues, promote carcinogenesis through direct impacts on the neoplastic transformation of epithelial cells [19; 20]. The involvement of oncogenic bacteria in cancer development will be discussed in the next section.

**Table 1:** Key bacterial species associated with gastrointestinal and extraintestinal cancers. Modified from [1]

Type of cancer	Microbiome between-species variation
Colorectal cancer	High abundance of *Fusobacterium*, *Selenomonas*, and *Leptotrichia* spp in cancer tissues.
Esophageal cancer	High abundance of *Streptococcus*, *Prevotella*, and *Veillonella* spp in cancer tissues.
Pancreatic cancer	High abundance of *Enterobacteriaceae*, *Pseudomonadaceae*, *Moraxellaceae*, and *Enterococcaceae* in cancer tissues.
Head and neck cancer	High abundance of *Gemella*, *Prevotella*, and *Fusobacterium* spp in cancer tissues. *Rothia* and *Streptococcus* spp were found at lower concentrations in cancer tissues.
Breast cancer	High abundance of *Prevotella*, *Veillonella*, and *Streptococcus* spp in cancer tissues.
Prostatic cancer	High abundance of *Propionibacterium acnes* in cancer tissues.

## Oncogenic bacteria and mechanisms of cancer development

**Fig. 2:**
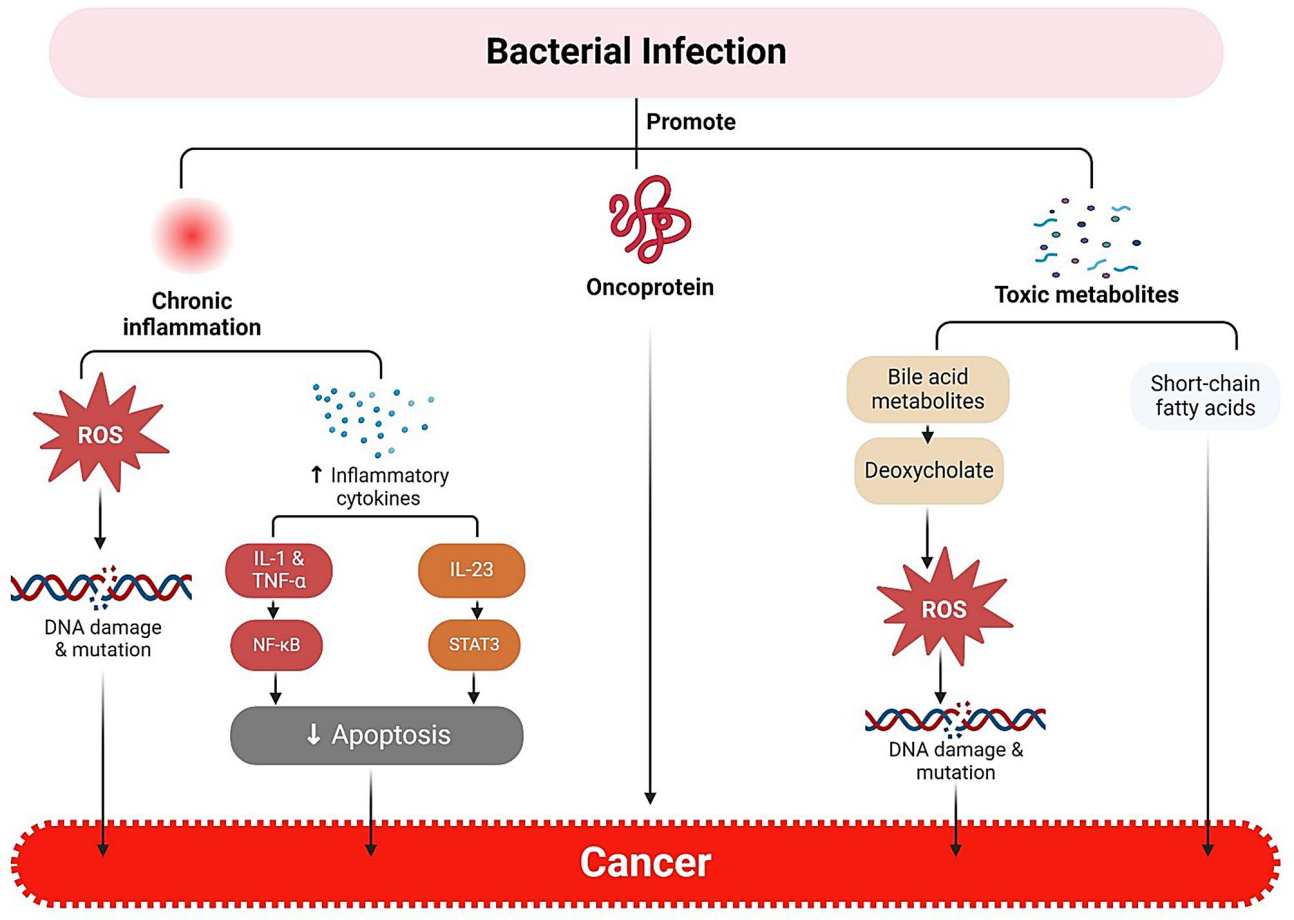
Different molecular pathways of cancer development triggered by bacterial infection. Recent investigations suggest that bacteria can directly modify host cell physiology via a variety of mechanisms, such as invasion and immune evasion, inflammation, altered cell signaling, initiation of DNA damage, and the formation of oncoproteins that cause mitogenesis or mutagenesis [23]. Created with BioRender

It is thought that cancer-causing microorganisms influence a number of immunological responses, which contribute to the growth of tumors. However, the exact process by which microorganisms cause cancer is still unknown. However, little is known about the bacteria-related variables that could affect neoplasia. The length of the infection (acute or chronic) affects carcinogenesis as well [21]. Recent research has demonstrated that inflammation plays a critical role in the development of tumor growth and that there is a direct causal relationship between the two [22] (Fig. 2). It was known that the tumor microenvironment, which is mostly controlled by inflammatory cells, plays a crucial role in the development of cancer by promoting the processes of migration, survival, and proliferation. Tumor cells have also appropriated innate immune signaling molecules for invasion, migration, and metastasis, including selectins, chemokines, and their receptors [24].

Enzymes, carcinogenic peptides, and bacterial toxins can all strongly contribute to the development of tumors by inducing inflammation that promotes ongoing cell proliferation and a higher chance of oncogenic transformation, obstructing cell cycle regulation, and upsetting cell signaling pathways [25]. Additional research has confirmed that the microbiome promotes the growth of cancer cells by attacking the DNA of the host cell, modifying the immune system, and encouraging the transition from epithelial to mesenchymal tissue [14, 26] (Table 2). Additionally, numerous genetic and epigenetic alterations that interfere with normal cell growth, survival, and control can lead to cancer. These alterations are influenced by a broad spectrum of internal and external variables. External factors include diet type, smoking/tobacco use, radiation, and pathogenic organisms; intrinsic factors include genetic mutations, random errors in DNA replication, immunological and inflammatory responses, and other modifiable factors, including aging [27].

**Table 2:** Cancer formation and carcinogenesis pathways are mediated by different microbiomes [23]

Associated Cancer Type	Bacterium	Proposed Oncogenic Mechanism
Gall bladder	*Salmonella typhi*	• Cytolethal distending toxin B (CdtB) • Biliary deoxycholate metabolite • p53 gene mutations • Protein kinase activation • Upregulation of the PI3K pathway
Lung cancer	*Chlamydia pneumoniae*	• Increased secretion of cytokines, IL-8, IL-10, and TNF • Overexpression of miRNA-328 activation of lung resident T cells • Synthesis of Myd88- dependent IL-1b and IL-23 • Production of reactive oxygen species (ROS)
Gastric carcinoma	*H. pylori*	•Mucosal barrier degradation caused by the apoptosis-stimulating protein p53 •Downregulation of heat shock protein1(HSP1) •Increases CDX1 expression, which promotes cell proliferation •Activation of the PI3K/Akt pathway
Colorectal cancer	*Streptococcus bovis*, *Enterococcus faecalis*, *E. coli*, *Bacteroides fragilis*, *Fusobacterium* spp., *Clostridium septicum,* and *H. pylori*	• *Bacteroides fragilis* toxin activates NF-κB that increase the expression of IL-17A and TNF-α • Catenin stimulate IL-17 R, NF-κB, and STAT3 signals • Induction of colibactin (clbB) and *Bacteroides fragilis* toxin (BFT) that increase colonic epithelial DNA damage
Breast cancer	• *Methylobacterium radiotolerans*, *Sphingomonas yanoikuyae*	• The microbiota secretes bioactive metabolites such as short-chain fatty acids, estrogens, secondary bile acids, or amino acid metabolites • Dysbiosis

### Gastric carcinoma

The stomach has traditionally been regarded as a sterile organ due to its acidic nature. Barry J. Marshall and Robin Warren’s discovery of *H. pylori* altered the paradigm [28], and more recently, DNA sequencing technology has verified the existence of additional bacteria from various phyla, such as *Proteobacteria*, *Firmicutes*, *Bacteroidetes*, *Actinobacteria*, and *Fusobacteria* in the stomach. It is now well known that the greatest risk factor for developing gastric adenocarcinoma is *H. pylori* infection as it is classified as a class I carcinogen by the WHO and is responsible for about two-thirds of all cases of stomach cancer globally [2] (Fig. 1).

**Fig. 3:**
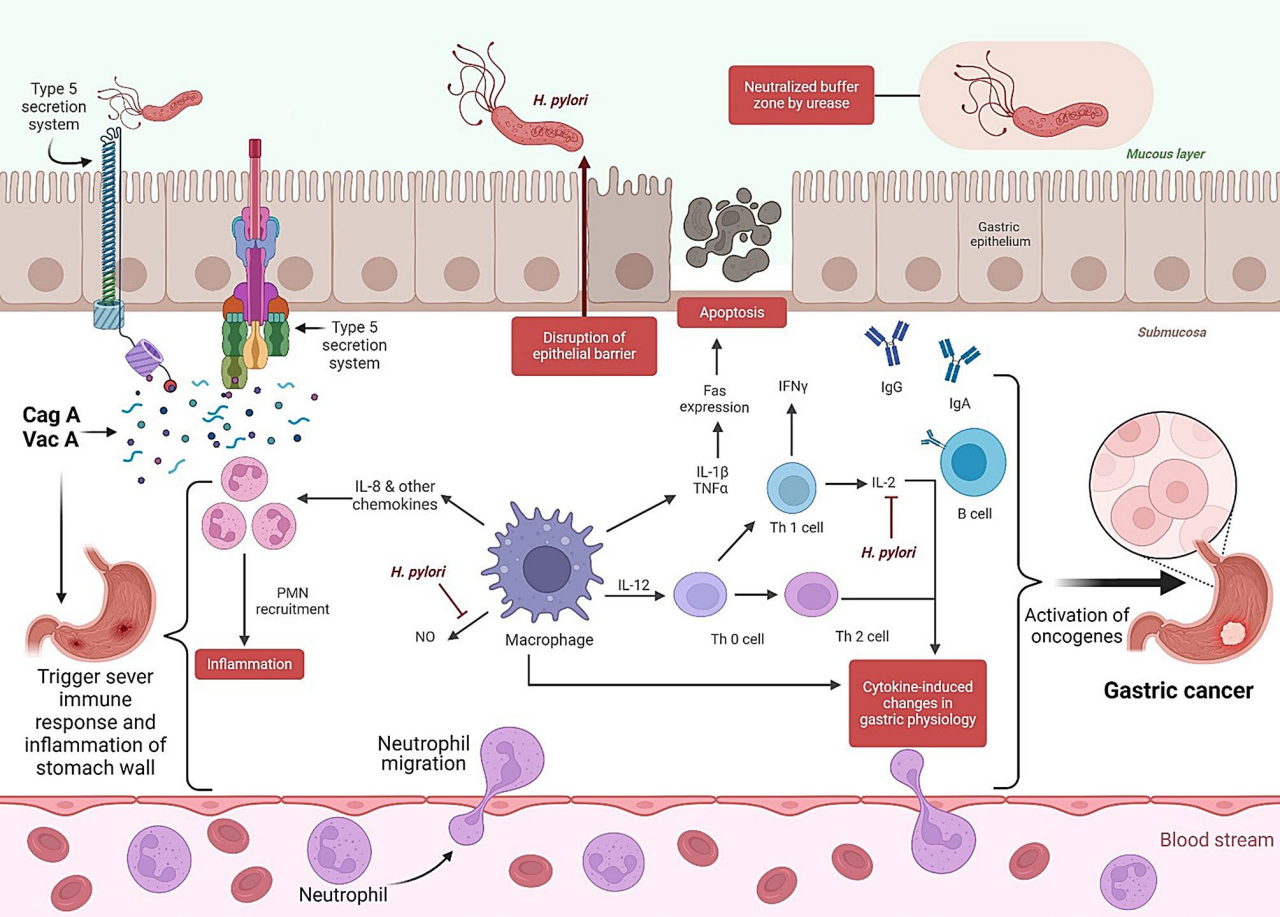
Etiopathogenesis of *H. pylori*-associated gastric cancer. Adapted from [13]. Created with BioRender

*H.* *pylori* is a gram-negative, microaerophilic spiral bacterium and a well-known oncomicrobe [19, 29]. *H. pylori* exists in several strains based on phenotypic variations and genetic differences. *H. pylori* isolates are frequently defined by the presence of a specific virulence gene known as cytotoxin-associated gene A (*CagA*) since gastric colonization with CagA-expressing strains raises the risk of peptic ulcers and is associated with a higher risk of gastric adenocarcinoma [29, 30]. *H. pylori* promotes oncogenesis through a number of mechanisms, including interactions between ROS and CagA and vacuolating cytotoxin A (VacA) (Fig. 3). CagA, VacA, and CagY are examples of promoters that affect gene expression, alter cell differentiation, or speed up cell division [31].

The initial stage of *H. pylori*‘s bacterial pathogenesis is the binding of the bacteria to host gastric epithelial cells and tissues through its adhesin HopQ. It then engages certain cellular carcinoembryonic antigen-related cell adhesion molecules (CEACAMs), which are necessary for the transfer of its virulence factor CagA via the type IV secretion system (T4SS) into the cytoplasm of host cells [32]. Following translocation into host cells, Src family kinases have the ability to phosphorylate CagA, which then binds SHP-2 in the cytoplasm. By activating the ERK–MAPK signaling pathway, CagA–SHP-2 complexes promote cell proliferation and prevent apoptosis. This results in an increase in the production of the anti-apoptotic proteins BCL-2 and BCL-Xl [18]. It was discovered that CagA interacts with the p53 protein to cause mutagenesis, which in turn triggers anti-apoptotic responses. CagA was revealed to have altered a large variety of proteins and pathways, such as anti-apoptotic factors of the B-cell lymphoma family, such as MCL-1, BCL-2, and BCL-Xl, and the kinases ERK and Akt [6].

Furthermore, there is a downregulation of autophagy and an increase in inflammation due to the repression of proapoptotic proteins such as BCL-2-like protein 11 (BIM), BCL2-associated agonist of cell death (BAD), and the apoptosis regulator SIVA1. It was recently shown that CagA may downregulate the SIVA1 protein through the PI3K/Akt pathway, which inhibits apoptosis and damages DNA [33]. A crucial CagA target and another human tumor suppressor, the apoptosis-stimulating protein of p53 2 (ASPP2), contributes to the survival of CagA-positive *H. pylori* in the lumen. Nevertheless, more research is needed to completely understand the molecular foundation causing the disturbance of gastric epithelial cell polarity seen in the aforementioned event and the ensuing oncogenesis [34].

The VacA proteins generate anion channels in cell membranes by assembling them into specific oligomeric complexes. The VacA-carrying strains, especially those with s1/m1 alleles, cause intracellular acid vacuoles in the stomach epithelial cells and are cytotoxic. They also affect the permeability of polarized epithelial cell monolayers, engage in immune system interactions, and induce programmed cell death in epithelial cells [6].

Increased oxidative stress-related harm in gastric cells is associated with *H. pylori* infection. It has been demonstrated that the release of chemokines, nitric oxide, and reactive oxygen species (ROS) by stomach epithelial cells infected with *H. pylori* triggers the generation of proinflammatory cytokines, such as interleukin-8 (IL-8) and IL-6. These cytokines have been demonstrated to possess an inflammatory function in both initiating and promoting the carcinogenic processes [35] (Fig. 3). IL-6 is essential in triggering several inflammatory signaling pathways, such as AMPK, MAPK, PI3K, JAK, and STAT3 [36].

Errors in replication and the build-up of mutations due to oxygen-free radical accumulation occur with an increasing rate of proliferation. In the DNA, guanine is converted to thiamine by 8-hydroxy-2’-deoxyguanine (8HdG), a by-product of oxidative damage caused by ROS [37]. It is discovered that the levels of this marker correlate with the infection, suggesting that the bacterium and its metabolite are both mutagenic. Thus, it is reasonable to believe that the inflammatory explanation of *H. pylori-*induced oncogenesis is correct [13].

More often found in gastric cancer sera are other virulence factors that contribute to the bacterial colonization of the gastric mucosa, such as adhesions and outer membrane proteins like outer inflammatory protein A (OipA), sialic acid-binding adhesin A (SabA), and blood group antigen-binding adhesin A (BabA). Furthermore, it has been discovered that BabA promotes the development of stomach carcinogenesis and double-stranded DNA (dsDNA) breaks. In addition to the previously described methods, *H. pylori* can inhibit tumor suppressor genes like E-cadherin and disrupt the methylation of DNA in gastric epithelial cells [13]. Additionally, prolonged use of PPIs and regular use of antibiotics can also lead to dysbiosis, which has been linked to a 2.4-fold increase in the overall risk of stomach cancer [18].

Additionally, *H. pylori* infection has been linked to mucosa-associated lymphoid tissue (MALT) lymphoma, a rare form of non-Hodgkin lymphoma caused by stomach B-cells. More than 90% of MALT lymphoma patients have *H. pylori* infection, and CagA plays a critical role in the pathogenesis [18, 38].

### Colorectal cancer

**Fig. 4:**
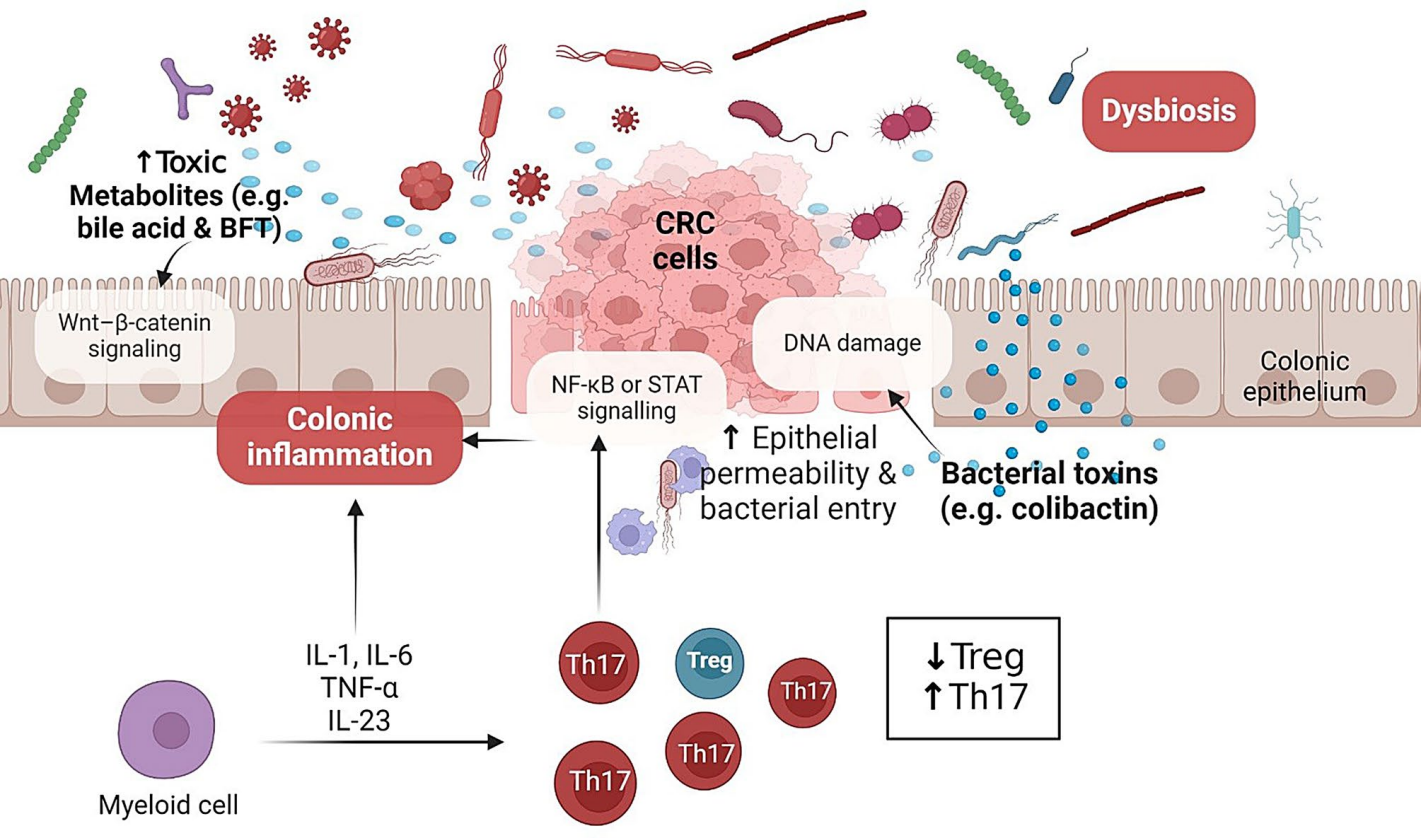
General mechanisms of bacteria-associated or -induced colon cancer. Dysbiosis and bacterial infection can initiate CRC by decreasing beneficial bacterial metabolites, increasing toxic bacterial products, disrupting tissue barriers and bacteria translocation, abnormal immune activation, and persistent inflammation, which lead to hyperproliferation and cancer development. Adapted from [40]. Created with BioRender

A number of alterations in the bacterial makeup of the gut microbiome have been linked to colorectal cancer, suggesting a significant role for dysbiosis in the development of colorectal carcinogenesis. Numerous bacterial species have been found and are thought to be involved in the development of colorectal cancer. These include *Fusobacterium spp., E. coli, Bacteroides fragilis, H. pylori, Enterococcus faecalis, Clostridium septicum, and Streptococcus bovis*. It has been discovered that there may be connections between the bacterial microbiome and colorectal carcinogenesis, including genotoxicity and bacterial-induced inflammation [39] (Fig. 4).

Several strains of *Bacteroides fragilis*, including enterotoxigenic and nontoxigenic *B. fragilis* (NTBF), have been found associated with colorectal cancer. By raising the numbers of T-helper cell 17 (Th17) and T regulatory (Treg) cells, *B. fragilis* is accountable for encouraging the development of colorectal cancer. The development of colon cancer is significantly influenced by the metalloprotease toxin of *B. fragilis*, which is an enterotoxigenic (ETBF). Actually, ETBF stimulates STAT3 signaling, which raises inflammation and increases the number of CD4-T cells that secrete IL-17 [6]. *B. fragilis* toxin (BFT) stimulates a procarcinogenic multistage inflammatory cascade in colonic epithelial cells (CECs) that include NF-κB, STAT3 signaling, and IL-17 R. Even though it is required, STAT3 activation in CECs is insufficient to result in ETBF colon carcinogenesis. Consequently, BFT triggers a Th17 mucosal response, which in turn prompts the CEC to initiate a procarcinogenic signaling cascade that preferentially activates NF-κB in distal colon CECs. This activation of NF-κB in distal colon CECs leads to myeloid cell-based carcinogenesis [12]. When comparing the colonic mucosa of familial adenomatous polyposis patients to that of healthy individuals, genes encoding secreted oncotoxins, BFT, and colibactin (clbB), were shown to be significantly enriched [41]

Ulcerative colitis (UC) and Crohn’s disease (CD) are two significant conditions that raise the risk of colorectal cancer. According to *Martin et al.* there are increased concentrations of some *E. coli* strains in inflammatory bowel diseases. They postulated that inflammatory bowel illness alters mucosal glycosylation, which may influence bacteria that cling to the mucosa and be involved in the etiology of Crohn’s disease, which raises the possibility of developing sporadic colon cancer [42].

Toxins include colibactin, cyclomodulin, cytolethal distending toxins (CDT), cytotoxic necrosis factor (CNF), and circulatory inhibitory factor (Cif) have been reported to be produced by pathogenic *E. coli* strains. These toxins can influence cell differentiation, proliferation, and apoptosis by interfering with the cell cycle and/or encouraging DNA damage [43]

When compared to mice treated with a single bacterial strain, tumors colonized with ETBF and E. coli demonstrated increased colonial IL-17 and colonic epithelial DNA damage, as well as earlier onset of tumors and higher mortality. This study found an improbable connection between tumor-causing microorganisms and early colon neoplasia.

Gastrointestinal diseases, particularly colon cancer, are linked to S. bovis. Gold et al. demonstrated that *S. bovis* pathogenic activity was limited to the colonic mucosa, the site of existing preneoplastic lesions. It appears that in an animal model generated by chemicals, the wall-extracted antigens (WEA) of the bacterium increase carcinogenesis [44]. According to Biarc et al. the Caco-2 cells produced prostaglandin E2 and chemokines (human IL-8 or rat CINC/GRO) in response to stimulation with either *S. bovis* WEA or cell-associated proteins (PgE2) [45].

PGE2 release in human cells is associated with an overexpression of cyclooxygenase-2 (COX-2), an enzyme that is essential for mucosal inflammation, apoptosis suppression, and angiogenesis stimulation. Furthermore, *S. bovis* proteins have the ability to activate MAPK kinases to promote proliferation. In the context of colorectal cancer, *S. bovis* has the ability to emit a particular “bacteriocin” that kills closely similar gut commensals and improves colonization of the mouse colon [6].

### Esophageal carcinoma

One of the most aggressive cancers and the sixth largest cause of cancer-related mortality is esophageal cancer [46]. It is the eighth most prevalent cancer type globally, with a 5-year survival rate of less than 25% [47].

Two of the primary bacterial risk factors for esophageal cancer are Barrett’s esophagus (BE) and gastroesophageal reflux disease (GERD). Lower microbial richness in the upper gastrointestinal tract has been linked to esophageal squamous dysplasia, which is thought to be a precursor lesion of esophageal squamous cell carcinoma [48]. Blackett et al. studied the microbiome of patients with BE, GERD, esophageal cancer, and reflux-free controls. They noticed that *Campylobacter* is noticeably more concentrated in the specimens from BE and GERD patients than it is in the specimens from controls. Furthermore, the expression of cytokines associated with carcinogenesis such as IL-18 is elevated in the presence of *Campylobacter*. Thus, based on the pathogenicity of *Campylobacter* species, it appears that *Campylobacter* may have a similar role to *H. pylori* in the development of stomach cancer in the progression of esophageal adenocarcinoma [49]. The carcinogenic risk of *Campylobacter jejuni* may result from the production of CDT, a genotoxin with DNase activity that may break down dsDNA [50].

The effect of penicillin G and streptomycin usage on the development of esophageal cancer was evaluated in an animal model of rats. The results indicated that the antibiotic treated group had a lower proportion of *Lactobacillales* and greater proportions of *Clostridium* than the control group. Nevertheless, such a change in the microbiome did not affect the incidence of esophageal adenocarcinoma [51].

When compared to normal epithelium, Zaidi et al. found that patients with BE and esophageal cancer had higher levels of *E. coli*. Additionally, it was shown that esophageal cancer had significantly increased expression of the TLR1-3, 6–7, and 9 signaling pathways, indicating a potential mechanism by which Escherichia coli might encourage cancer development of esophageal adenocarcinoma [52].

It’s unclear exactly what part the microbiome plays in esophageal systemic sclerosis (SSc). *Yu et al.* proposed that those with a reduced esophageal microbiome may be more susceptible to esophageal squamous cell dysplasia [53]. However, Nasrollahzadeh et al. discovered that the gastric corpus microbiome of patients with SSc and esophageal squamous cell dysplasia was primarily made up of *Clostridiales* and *Erysipelotrichales* in contrast to healthy controls. This implies a potential role for abnormalities in the stomach microbiome in the conversion of esophageal squamous dysplasia to SSc [54]

Furthermore, it has recently been shown that patients with esophageal squamous cell carcinoma (SCC) have a much higher prevalence of *Porphyromonas gingivalis* in their saliva and neighboring mucosal tissues than in healthy controls [2] Meng et al. reported that *P. gingivalis* increased SCC cell line proliferation and motility through the NF-κB signaling pathway. These results point to the involvement of oral infections in the development, metastasis, severity, and unfavorable prognosis of esophageal SCC [55].

It has recently been shown that there may be a relationship between the prognosis of esophageal SSc and the presence of *Fusobacterium nucleatum*. *F. nucleatum* was discovered in the tissues of esophageal cancer of 74/325 patients, or around 23% of the total, and it was strongly associated with a shortened survival period. Activation of the cytokine–cytokine receptor interactions by *F. nucleatum* may induce an aggressive tumor phenotype, as evidenced by increased gene expression of the particular chemokine CCL20 [2].

### Lung cancer

The lung, as a barrier site that interacts with the outside environment with each breath, is vulnerable to local inflammation caused by environmental allergens, viral exposures, toxins, and cigarette smoke. Although chronic inflammation and lung cancer are closely associated, the fundamental immunological mediators and causes of inflammation remain unclear. Non-small cell lung cancer (NSCLC), the most prevalent kind of lung cancer, is the largest cause of cancer-related deaths globally, therefore understanding the roles of all elements that contribute to its carcinogenesis and treatment response is critical for public health [56]. Furthermore, it is becoming clear that the lung microbiome may have a role in lung cancer [57] (Fig. 4). The precise role of the lung microbiome in NSCLC is still unknown, and some research shows that few viable microbes can be recovered from healthy lungs, perhaps due to low biomass or technological detection limits. However, a history of bacterial pneumonia or another lung infection is present in over 50% of NSCLC patients [58–60].

Approximately 10% of cases of community-acquired pneumonia are caused by Chlamydophila [61]. There has been evidence of *C. pneumoniae* in 230 cases of lung cancer, and those with *C. pneumoniae* infection had a 1.6-fold greater chance of developing lung cancer. It has been discovered that individuals with lung cancer who had *C. pneumoniae* infections have higher levels of IgA antibodies [23]. In line with this, a team of scientists performed a meta-analysis of 12 published papers that looked at the connection between lung cancer and *C. pneumoniae* infection. The study found a correlation between *C. pneumoniae* infection and an increased risk of lung cancer, suggesting that a higher serological titer could be a useful indicator of the likelihood of developing lung cancer [62]. A recent systematic assessment of published articles on the relationship between lung cancer development and *C. pneumoniae* infection was carried out. Twenty-four papers describing one animal model favorably supported the concept, while three pieces did not, according to the study’s authors [63].

Many proteins secreted after chlamydial infection are thought to promote lung cancer by interfering with cytoplasmic or mitochondrial cellular functions. These targeting proteins and host proteins compete with one another to bind to the substrate through a process known as competitive inhibition. Lung cancer develops as a result of this disruption of regular cellular growth, which changes apoptosis, or programmed cell death [64]

Gene damage and neoplastic transformation are two additional pathways of bacterial infection that cause lung cancer, which are activated by ROS, nitric oxide (NO), and inflammatory mediators. Human alveolar macrophages and peripheral blood mononuclear cells secrete more IL-8, IL-10, and TNF when they are chronically infected with *C. pneumoniae*, according to an in vitro investigation. Human NSCLC development is stimulated by the angiogenic factor IL-8. In addition, NO release is elevated in chlamydial infection, which may trigger an inflammatory reaction that results in the growth of lung cancer [23].

The generation of IL-1b and IL-23 from myeloid cells, which are dependent on Myd88, has been discovered to be promoted by the microbiome. This, in turn, leads to the proliferation and activation of γδ T cells, which carry IL-17 and other effector chemicals that promote inflammation and tumor cell proliferation. Furthermore, some taxa are associated with oncogenic transcriptome programs in NSCLC tissues, such as the activation of the phosphoinositide 3-kinase (PI3K) and ERK signaling pathways. This was further confirmed by in vitro and in vivo exposure of airway epithelial cells to bacteria, including *Streptococcus*, *Veillonella*, *and Prevotella*, which activate PI3K–AKT signaling. Increased chlamydial infections, which may trigger an inflammatory response that results in the development of lung cancer, have been linked to the development of lung tumors through the correlation of local microbiome-immune crosstalk. Additionally, molecular mediators that can be useful targets for lung cancer intervention have been identified [65].

### Gall bladder cancer

Gall bladder cancer (GBC) is the most frequent kind of cancer of the biliary system, yet it is a rare cancer overall. This is a particularly aggressive cancer with significant geographic variability and a high tendency for metastasis [66]. Persistent parasitic and bacterial infections raise the risk of GBC [67]. More specifically, there has long been a correlation between *Salmonella* infections and GBC, especially *S. typhi* [68]. Thus, GBC is considerably more common in regions where typhoid is endemic, such as northern India [69] and Chile [70].

*S.typhi* has a role in triggering malignant transformation by altering the genomic sequence of tumor protein p53 (TP53) and amplification of the protooncogene c-MYC in susceptible mice gall bladder organoids and fibroblasts. When a mitogen and the Akt pathways cause the autoactivation of protein kinase, *S. typhi* effectors secreted during an infection play a part. It has been consistently observed in gallbladder cancer patients that this mechanism is pathognomonic in both commencing and sustaining malignant transformation. Thus, it was determined that *S. typhi* predisposed the gallbladder’s epithelium to harmful metabolites [16].

*S.typhi* is a powerful oncomicrobe in GBC [71]. *S. typhi* produces its virulence component, AvrA, through its type 3 secretion system, which triggers the JAK-STAT pathway and Wnt–β-catenin signaling. The synthesis of genotoxic chemicals by *S. typhi*, specifically cytolethal distending toxin B (CdtB)—the functional unit of CDT and cytotoxic necrotizing factor 1 (CNF1)—induces dsDNA breaks through its DNase-like activity [72; 73]. While CNF1 blocks the function of cytokines that cause inflammation and cell cycle suppression, CdtB targets the DNA in the human host cells to achieve its desired effect. Moreover, CNF1 affects the transcription termination process in prokaryotes by modifying the Rho proteins (Sheweita et al. 2020).

Toxic metabolites and secondary bile acids are produced in large quantities by *S. typhi* principally by its metabolism of bile acids. The gall bladder’s epithelium becomes pathological as a result of these harmful chemicals. The conjugated primary bile salts are deconjugated by the glycosidase enzyme β-glucuronidase, which produces highly toxic compounds with carcinogenic properties in high quantities [74] These metabolites exert their mitogenic effect via binding to DNA in human epithelial cells [75].

A different theory suggested that *S. typhi*‘s mutagenicity results from its interaction with cholesterol, which eventually serves as the structural basis for gallstones. Subsequently, *S. typhi* causes harmful changes in epithelial cells by turning cholesterol into carcinogenic compounds such as 5-alpha, and 6-epoxide cholesterol and transforming bile salts into secondary bile form [23].

### Breast cancer

Breast cancer is the most frequent type of cancer in women. Worldwide, breast cancer is one of the leading causes of death for women. One in eight women will have breast cancer in their lifetime [23].

One of the hallmarks of breast cancer is dysbiosis, which is an abnormal microbiome composition. Bioactive metabolites (short-chain fatty acids, reactivated estrogens, secondary bile acids, or amino acid metabolites) secreted by the gut microbiome are known to influence breast cancer. It has been demonstrated that these blood-borne microbial metabolites alter the behavior of breast cancer. Since these metabolites originate in a “gland” (the microbiome in this example) and are subsequently transported through the bloodstream to distant locations of action, they bear resemblance to human hormones. These metabolites are frequently vital components of the tumor microenvironment [15].

**Fig. 5:**
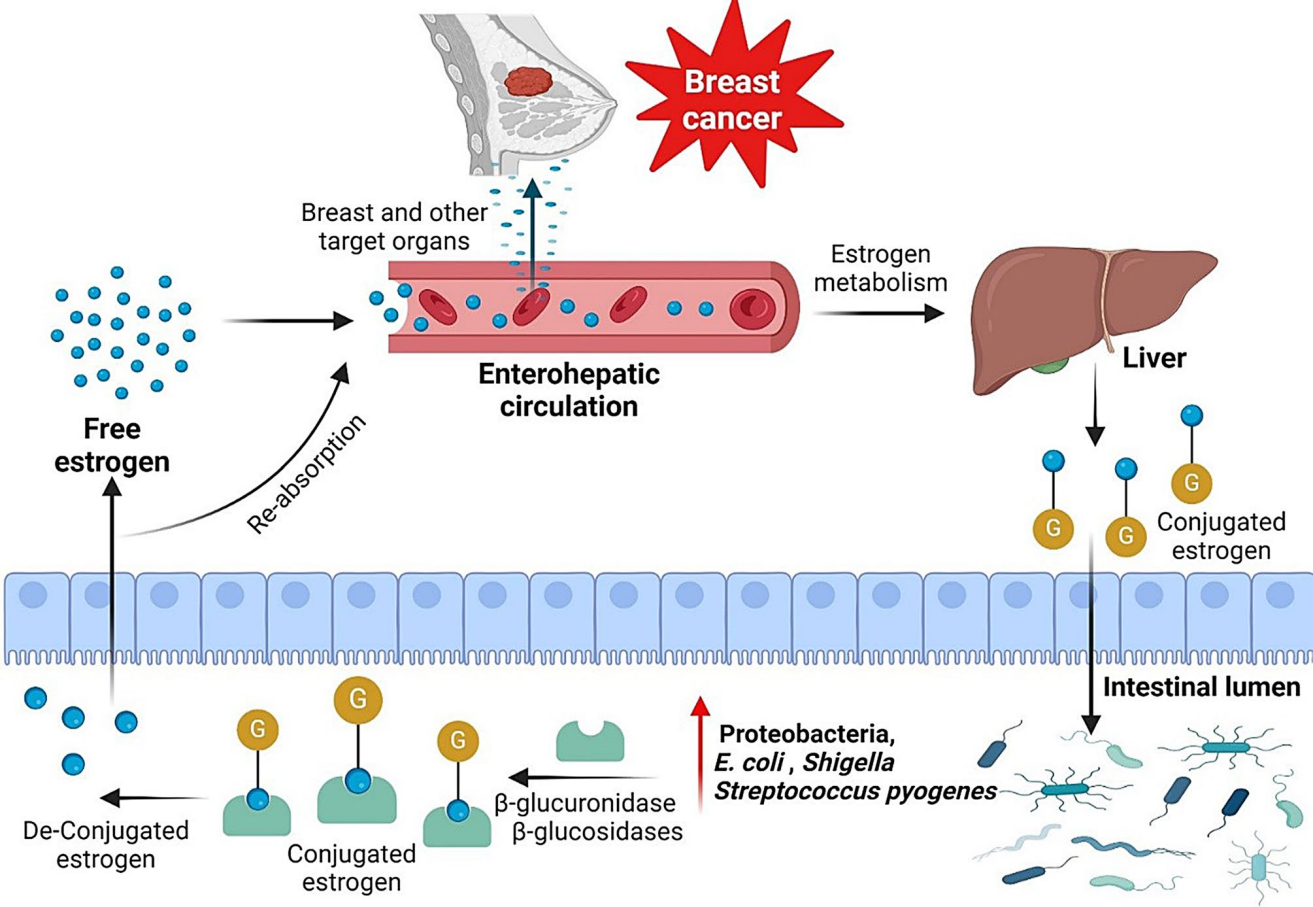
Role of dysregulated bacterial communities in breast cancer oncogenesis. In dysregulated bacterial communities, certain bacteria (such as *Proteobacteria*, *E. coli*, *Shigella*, and *Streptococcus pyogenes*) produce estrogen-metabolizing enzymes (β-glucuronidase and β-glucosidases) which deconjugates conjugated estrogen metabolites. Consequently, they can be reabsorbed as free estrogens via the enterohepatic circulation and taken up by different organs, including the breast. (created with BioRender)

According to a number of recent research, some bacteria may contribute to breast cancer. Estrogen-metabolizing enzymes produced by distinct bacterial genes can adjust the levels of estrogen in the bloodstream. For instance, β-glucuronidase, which deconjugates with metabolites that resemble estrogen, is produced by a number of bacteria, including those in the Ruminococcaceae and Clostridium families. Because of this, they can be reabsorbed as free estrogens through the enterohepatic circulation and absorbed by many organs, the breast is one of them. Estrogen-like substances have two distinct effects: they can either promote the growth of particular bacteria or trigger the manufacture of growth factors that are stimulated by estrogen, which may have carcinogenic potential. In situations when there is ongoing, persistent, and dysregulated inflammation, bacteria may exacerbate breast cancer [6] (Fig. 5).

Emerging evidence suggests a biological link between gut and breast cancers mediated by shared microbial signatures and microbiome-driven metabolic pathways. Several bacterial genera commonly associated with gastrointestinal dysbiosis—such as *Bacteroides*, *Streptococcus*, and other taxa—have been detected at altered abundance in breast tissue from cancer patients compared to healthy controls, indicating possible microbial translocation or systemic influence linking gut and breast microbial ecology [76–78].

Moreover, the gut microbiome plays a central role in regulating systemic estrogen metabolism through the so-called “estrobolome,” a community of gut bacterial genes capable of producing β-glucuronidase and other enzymes that deconjugate estrogens, allowing reabsorption via the enterohepatic circulation. Dysregulation of the estrobolome may alter circulating estrogen levels and contribute to hormone-receptor–positive breast cancer risk [79].

In addition, gut-derived microbial metabolites such as short-chain fatty acids (SCFAs) and bile acid derivatives can enter systemic circulation and influence immune regulation, epithelial proliferation, and inflammatory signaling in distant tissues including the breast. Such metabolic crosstalk provides a plausible mechanistic link between gut dysbiosis and breast tumorigenesis [80, 81].

Clinical and experimental studies further support this connection: analyses of gut microbiota composition in breast cancer patients reveal altered diversity, enrichment of certain bacterial taxa, and associations with metabolic and hormonal biomarkers [82, 83].

Collectively, these findings support a mechanistic correlation between gut and breast cancers, whereby gastrointestinal microbial dysbiosis, through altered estrogen metabolism, systemic metabolite flux, immune modulation, and microbial translocation, may contribute to breast carcinogenesis.

### Cervical carcinoma

Cervical cancer is fourth in terms of both incidence and mortality as of 2018, accounting for around 8% of cancer-related deaths. Numerous in vivo and in vitro investigations have shown a link between chlamydial infection and cervical neoplasia. Oncogenesis is thought to result from ongoing inflammation and metaplasia, especially from squamous cell metaplasia, however, the exact mechanism is yet unknown. It has been reported that *C. trachomatis* infection may lead to cancer, which can grow over long periods of time [13].

It is known that *C. trachomatis* infection alters the transcription of genes involved in cell differentiation, expression of transcription factors, and cell death. Mutagenesis is facilitated by altered metabolite production, prolonged inflammatory response, increased cytokine activity, and weakened cell-mediated immunity. These events destroy DNA repair mechanisms, accumulating abnormal DNA, and promote unregulated, multipolar mitosis subsequently leading to cancer [84].

Mitochondrial cytochrome C inhibition is another mechanism by which apoptosis is inhibited. Nonetheless, three distinct approaches to accomplishing this have been postulated. First, by producing anti-apoptotic substances that impede upstream actions regulating mitochondrial function. Second, although BCL-2 expression does not ensure that apoptosis is blocked, it may inhibit the activation of caspase and the synthesis of cytochrome c. Finally, relevant conjectures suggest the presence of other anti-apoptotic agents that are not yet known or understood [13].

During *C. trachomatis* infection, the tyrosine phosphorylation of host cell proteins involved in signal transduction circuits increases. Furthermore, *C. trachomatis* is shown to activate the carcinogenic constituents of the Ras-Raf-MEK-ERK cascade in addition to producing ROS in order to survive. The cytoplasm of cells contains the protein-folding enzyme heat shock protein-60 (HSP60). Like *E. coli*‘s GroEL, the HSP-60 can cause inflammation. *C. trachomatis* HSP60 may serve as an oncogenic risk factor by modulating apoptosis. The chlamydial HSP60-affected host cells are very susceptible to the production of oncogenes, continued proliferation, and eventually cancer development [85].

## Therapeutic strategies targeting Bacteria-Associated Carcinogenesis

Growing evidence demonstrates that bacterial pathogens, microbial toxins, and gut dysbiosis play a major role in initiating or promoting carcinogenesis. This has emphasized the need for therapeutic strategies targeting oncogenic bacteria and microbiome-driven pathways. Recent studies highlight the promise of targeted antimicrobial therapy, bacteriophage-based interventions, toxin-neutralizing agents, microbiome modulation, immunotherapy–microbiome synergy, and dietary approaches in reducing bacterial carcinogenic potential [86–91].

Therapeutic approaches against oncogenic bacteria can be broadly classified into five categories: (1) eradication of established carcinogenic infections, such as *H. pylori* and chronic *Salmonella Typhi* carriage; (2) neutralization of bacterial toxins and virulence factors, including colibactin and Bacteroides fragilis toxin; (3) microbiome modulation through probiotics, prebiotics, and dietary fiber to restore beneficial microbial balance; (4) emerging biologics, such as bacteriophages and CRISPR-based bacterial elimination; and (5) augmentation of anticancer immunity through manipulation of gut microbial composition, which enhances responses to immune checkpoint inhibitors. A recent review highlighted the expanding potential of microbiome-targeted therapeutics, including phage therapy and engineered microbial consortia, in regulating inflammation and suppressing tumor-promoting bacteria [86]. Another updated publication demonstrated that microbial dysbiosis contributes to tumor microenvironment remodeling and that probiotic intervention can reduce pathogen-induced inflammation [87]. Meanwhile, new small-molecule inhibitors targeting bacterial genotoxins and quorum-sensing pathways have been developed and tested in vivo, showing reduced DNA damage and tumorigenic progression [88]. Additionally, systems biology studies confirm that targeting microbiome–immune interactions can suppress cancer-associated inflammation and improve therapeutic response [89].

Together, these findings emphasize that targeting bacteria-associated carcinogenesis requires an integrative therapeutic framework addressing microbial eradication, bacterial activity neutralization, immune modulation, and microbiome ecology restoration. The following Table 3 summarizes major therapeutic strategies, their mechanisms of action, targets, and supporting evidence from recent high-impact publications.

**Table:** 

Therapeutic Approach	Mechanism of Action	Target (Bacteria/Pathway)	Supporting References
Standard Eradication Therapy	Antibiotic-based elimination of carcinogenic bacteria	*H. pylori*, *Salmonella Typhi* (chronic carriage)	[92–98]
Anti-Toxin & Virulence Factor Inhibitors	Inhibit bacterial toxins (colibactin, BFT), block virulence and DNA-damage pathways	pks^+^ *E. coli*, ETBF	[88]
Microbiome Modulation (Probiotics, Prebiotics, Synbiotics)	Restores eubiosis; strengthens epithelial barrier; reduces inflammation	Dysbiosis, loss of SCFA-producers	[87, 90]
Bacteriophage Therapy	Phage-mediated selective elimination of tumor-promoting bacteria	*Fusobacterium*, pathogenic *E. coli*	[86]
CRISPR-Based Precision Antimicrobials	Gene-specific removal of virulent bacterial strains	pks island, virulence genes	[99–101]
Immunotherapy–Microbiome Synergy	Modulates microbial taxa to enhance immune checkpoint inhibitor response	Tumor-associated microbiota	[89]
Dietary Interventions (High Fiber, Polyphenols)	Boosts SCFA production; reduces inflammation; modulates gut ecology	Dysbiosis; SCFA deficiency	[102]
Fecal Microbiota Transplantation (FMT)	Replaces dysbiotic microbiome with healthy ecology	Post-antibiotic dysbiosis; therapy resistance	[102]

Therapeutic strategies against bacteria-driven carcinogenesis now extend far beyond conventional antibiotic eradication and increasingly rely on precision approaches targeting specific microbial mechanisms. Standard eradication therapy remains essential for pathogens with established carcinogenic roles, such as *H. pylori* [92–96] and chronic *S. Typhi* carriers, where antibiotic treatment demonstrably reduces the risk of gastric and gallbladder cancers [97, 98]. However, emerging evidence shows that simple eradication may be insufficient because oncogenic pathways often persist through virulence factors and inflammatory signaling.

Growing insight into bacterial genotoxins has driven the development of targeted inhibitors that neutralize microbial virulence rather than indiscriminately killing bacteria. Novel small-molecule inhibitors have been shown to block colibactin biosynthesis in pks^+^ *E. coli*, reducing DNA double-strand breaks and mutagenesis. Other compounds inhibit Bacteroides fragilis toxin (BFT), thereby preventing IL-17–driven inflammatory signaling and β-catenin activation [103–105]. Jayaprakash et al. provided an evidence that these agents suppress tumor-promoting bacterial activity without broad microbiome disruption, representing a promising shift toward mechanism-specific bacterial therapeutics [88].

**Microbiome-directed therapies**, such as probiotics, prebiotics, and synbiotics, have shown substantial promise in restoring beneficial microbial taxa, suppressing inflammation, and preventing tumor-associated dysbiosis [90]. Recently, Plewa et al. provided strong experimental support for the anti-inflammatory and anti-tumorigenic effects of probiotic supplementation, positioning microbiome modulation as a cornerstone for preventing cancer-promoting microbial imbalances [87].

**Bacteriophage therapy** is emerging as a selective and highly precise strategy for eliminating tumor-promoting bacteria such as *Fusobacterium nucleatum*, which plays a key role in colorectal carcinogenesis and chemoresistance. Phages can disrupt biofilms, reduce bacterial load, and inhibit virulence pathways with remarkable specificity. Luo et al. demonstrated that the success of phages in targeting bacteria implicated in tumor progression, noting their advantages over antibiotics—particularly in preserving beneficial commensal species and avoiding dysbiosis [86].

**CRISPR-Cas antimicrobial systems** offer next-generation precision by selectively eliminating pathogenic strains carrying oncogenic determinants such as colibactin gene clusters, biofilm regulators, or antibiotic-resistance elements. These tools can target bacteria at the genetic level without disturbing non-pathogenic microbiota, making them ideal for tackling polymicrobial communities implicated in cancer development. Preclinical studies show successful CRISPR-mediated removal of virulent *E. coli* strains and modulation of tumor microenvironment–associated microbes [99–101].

Additionally, **microbiome–immunotherapy synergy** has become one of the most clinically relevant therapeutic intersections, as gut microbial composition profoundly influences response to immune checkpoint inhibitors. The recent systems-biology analysis demonstrated that manipulating the microbiome can reprogram immunotherapy outcomes, highlighting the therapeutic importance of microbial ecology [89].

Finally, dietary interventions, including high-fiber and polyphenol-rich diets, and fecal microbiota transplantation (FMT) have shown benefits in restoring microbial balance, increasing SCFA production, and reducing inflammation—thereby attenuating microbial contributions to tumorigenesis [102].

## Conclusion

Bacteria-associated carcinogenesis is now recognized as a significant driver of cancer development across multiple organ systems. The evidence summarized in this review demonstrates that pathogenic bacteria contribute to tumor initiation and progression through mechanisms such as DNA damage, toxin production, chronic inflammation, immune modulation, metabolic reprogramming, and disruption of epithelial integrity. Beyond individual pathogens, dysbiosis and microbiome imbalance play crucial roles in shaping the tumor microenvironment and influencing systemic cancer behavior, including metastasis and therapeutic response.

Growing data linking gut microbiota to extraintestinal cancers, including breast and cervical malignancies, highlights the broader impact of microbial ecosystems on host physiology. Understanding these interactions provides new opportunities for biomarker discovery and targeted interventions. Emerging therapeutic strategies—such as bacteriophage therapy, toxin inhibitors, CRISPR-based antimicrobials, probiotics, dietary modulation, and microbiome-immunotherapy synergy—offer promising avenues for mitigating bacterial contributions to carcinogenesis.

Collectively, these insights underscore the importance of integrating microbiome research into cancer biology. Continued investigation into microbial mechanisms, tumor-resident microbiota, and host–microbe crosstalk will be essential to translate these findings into effective diagnostic, preventive, and therapeutic strategies. As the field evolves, microbiome-informed oncology holds the potential to transform cancer risk assessment, enable earlier detection, and enhance personalized cancer care.

## Data Availability

Not Applicable
